# Assessment of the implementation of community-led total sanitation, hygiene, and associated factors in Diretiyara district, Eastern Ethiopia

**DOI:** 10.1371/journal.pone.0175233

**Published:** 2017-04-13

**Authors:** Roba Argaw Tessema

**Affiliations:** Department of Environmental Health Sciences, College of Health and Medical Sciences, Haramaya University, Dire Dawa, Ethiopia; Centre for Injury Prevention and Research, BANGLADESH (CIPRB) & Örebro University, SWEDEN

## Abstract

Based on the sustainable development goals, the United Nations plans to achieve equitable sanitation and hygiene for all and to end open defecation by 2030. In Ethiopia, 60% to 80% of health problems are due to communicable diseases attributable to unsafe water supply, unhygienic and unsanitary waste management, which are directly linked to the practice of open defecation. This study has aimed at assessing the implementation of community-led total sanitation and hygiene (CLTSH) and associated factors. A community-based cross-sectional study design involving 420 of the 7,225 households found in Diretiyara district was conducted in June 2014. Both quantitative and qualitative data were collected. Using Logistic Regressions, bivariate and multivariate analyses were computed. This study showed that 66% of the respondents have knowledge of CLTSH. Eighty-nine percent of the respondents have latrine, of which 78% were constructed after the introduction of CLTSH. Eleven percent of the respondents reported to have defected in the open field and 15% of them reported that they had been recently exposed to diarrhea diseases. The occurrence of diarrheal disease was significantly associated with the extent of latrine ownership [AOR = 2.48; 95% CI 1.00, 6.12]. Attitude and perception parameters were significantly associated with consistent latrine utilization. Respondents who agreed that "Open defecation is preferred due to the unpleasant smell and heat from the Latrine'' [COR = 0.58; 95% CI 0.34, 0.99] were 58% less likely to use the latrine consistently. In conclusion, CLTSH has increased the extent of latrine ownership and decreased practice of open defecation, and yet, intermittent latrine use and poor hygienic practice were reported. Although some fundamental misconceptions were reported, the majority of the respondents have accepted CLTSH approach as a means to ending open defecation in their village. Health extension workers and local authorities should give emphasis to achieving sustainable behavioral change on improved sanitation and good hygiene practices.

## 1. Introduction

Diarrhea is the leading cause of child death in Africa and it is the second leading cause of child death globally [1]. Worldwide, 88% of the cases of diarrhea are attributable to unsafe water, inadequate sanitation and insufficient hygiene [2]. The magnitude of the sanitation related deaths is striking; 1.5 to 2.2 million people are estimated to die each year from diarrhea and related diseases and the great majorities are children [2, 3]. The strong links between these deaths and defecation in open spaces, lack of access to safe disposal of human excreta, lack of awareness of hygienic practices and using contaminated water are not disputable, and more than 7 out of 10 of the rural inhabitants are do not have improved sanitation [3].

To meet the Sustainable Development Goals (SDG) (Goal 6 target 2) by 2030, United Nation has planned to achieve access to adequate and equitable sanitation and hygiene for all and to end open defecation by paying special attention to the needs of women and girls and those in vulnerable situations [4]. Poor water, sanitation and hygienic (WASH) conditions are among the major causes of public health problems in Ethiopia, where children are the most vulnerable [5]. In Ethiopia, health statistics indicates that communicable disease accounts for about 60% to 80% of health problems which are preventable and considerable proportions of these diseases are directly related to unsafe water, poor hygiene and inadequate sanitation [6, 7]. The average Ethiopian children suffers 5 to 12 diarrhea episodes a year resulted from poor WASH and between 50, 000 to 112,000 under-five children die annually [8, 6, 9].

Although Ethiopia has substantially reduced Open Defecation (OD) from 92% (44.3 million) in 1990 to 29% (28.3 million) in 2015 [10, 11, 12], a considerable proportion of population still practices OD [12]. For low-income countries like Ethiopia, a cost-effective community-based participatory approach is essential to combat diseases caused by inadequate WASH [13]. In Ethiopia, CLTSH has remained the only instrument to ignite community-wide behavioral change and collective action to move the entire community toward improving sanitation [11, 14]. This approach focuses on eradicating OD at a community level by generating sustained behavioral change leading to spontaneous and long term abandonment of OD practices and stimulating demand for latrines without any external hardware support [11, 3, 15]. It enables the communities to conduct their own sanitation profile through appraisal, observation, and analysis of their practice of OD and its effects and the heart of this approach lies on having a paradigm shift from providing sanitation facilities to achieving collective behavioral changes with growing evidence on open defecation and creating open defecation-free (ODF) environments [3, 16].

Even if CLTSH has been implemented in over 50 countries, including Ethiopia [15], data on level of implementation of CLTSH and associated factors are scanty and there is a lack of rigorous and objective data, particularly in the study area and Ethiopia at large. Therefore, the present study is designed to assess the level of Implementation of Community-Led Total Sanitation, Hygiene and associated factors in Diretiyara district, Eastern Ethiopia.

## 2. Materials and method

### 2.1 Study area and population

Based on health and health-related indicator data, the Harari regional state has a total population of 220,000, of which 111,048 were males and 108,952 were females [17]. Fifty-five percent of the population lives in urban areas. The annual growth rate was 2.6%. The potential health service coverage was 100%. Latrine coverage of the region was 81% (86% being the national coverage) [17]. According to 2012 health and health-related indicator data, access to safe drinking water supply in the region was 75.8% (where 87% of the population lives in rural areas and 97% are urban dwellers). The under-five mortality rate is 94 per 1000 [18].

The region has nine districts, of which six (Amirnur, Abadir, Shenkor, Aboker, Janela and Hakim) are urban and three (Errier, Diretiyara, and Sofi) are rural. The CLTS was implemented in three rural districts, from which Diretiyara district was randomly selected. Diretiyara is 12kms away from Harar city. It is bordered with the Harari city in the east, Kombolcha on the west, Awoday in the south and Ijegina on the north. The district has a total of 7,225 households (with a population of 31,071). It has one health center, six health posts, eight elementary and junior (1–8) schools [19].

### 2.2. Study design and period

A community-based cross-sectional quantitative study was conducted in Diretiyara district in June 2014. The participatory qualitative methods were also conducted using Focus Group Discussion (FGD) and an observational check list.

### 2.3 Source, study and sample population

All the three districts (Errier, Diretiyara, and Sofi), which are found in rural areas where the CLTSH was implemented, were considered as source population. All the households found in Diretiyara district were considered as the study population. A total of 422 households were sampled from the study area, and all sampled household wives or husbands or a household member with age above 18 years was eligible for the interview and was taken as a sample population.

### 2.4 Sample size determination and sampling techniques

The sample size was determined by using a single population proportion formula:

$$n=\frac{z_{1-\alpha /2}^{2}*\left(P\left(1-P\right)\right)}{d^{2}}$$ (1)

$$n=\frac{1.96^{2}*\left(0.5\left(1-0.5\right)\right)}{0.05^{2}}=384hhs$$

By considering the desired precision of (d^2^) 5%, a 95% (Z1-∞/2) with confident level of 1.96 and P of 0.5, the final sample size was 422 households (including 10% non-response rate). Three FGDs were conducted with community leaders, model families, and health extension workers by supervisors. The systematic random sampling technique was used, and every 17^th^ (7225/422) household was eligible for interview. The simple random sampling technique was used to select a random start.

### 2.5 Study variables

#### 2.5.1 Dependent and independent variable

Knowledge about CLTSH, acceptance of CLTSH, attitude towards CLTSH, latrine availability, latrine consistent utility and practice of open defecation were taken as response variables. Socio-demographic and economic variables (age, occupation, marital status, religion, educational level, and monthly income), the practice of transient walk, feces mapping, feces calculation and feces mobility chart were considered as explanatory variables.

### 2.6. Data collection method and instruments

For quantitative data, fourteen data collectors who completed 12^th^ grade and who knew local languages and with previous experience in data collection, were involved after two days training and for qualitative data, two supervisors who have BSc in environmental health have participated. A structured questionnaire, prepared in Afan Oromo, was used. After one-day training was delivered to supervisors on how to administer data collection, FGD, with eight discussants at a time, was conducted fully.

### 2.7. Data quality control

Data collectors were trained for two days. The questionnaire was prepared in English language and then translated into Afan Oromo for ensuring its consistency. The questionnaires were pre-tested in other kebele to avoid any contamination of data. Supervision and checking of filling questionnaires were made on a daily basis in the field. (“*Kebele”* is the smallest administrative unit in Ethiopia.)

### 2.8. Data analysis

An Analysis was made by using the SPSS Version16 statistical package. Bivariate analyses were employed to examine the relationships between outcomes and explanatory variables and multivariate analyses were used to adjust for possible confounding factors. The odds ratio was computed to assess the strength of the association between response and explanatory variables. The significance of statistical associations was assured using odds ratio, a 95% CI and P Value. It was considered statistically significantly associated when the P value is less than 0.05 (*).

### 2.9. Operational definitions

Community-Led Total Sanitation and Hygiene (CLTSH) is a community-based approach that focuses on eradicating open defecation by generating behavioral change in sanitation at a community level and by stimulating demand for latrines and hygiene practice.

Knowledge about CLTSH steps: Respondents who explain at least three steps of CLTSH (pre-triggering, triggering, post-triggering and, scaling up and going beyond for sanitation ladder)

Knowledge about CLTSH tools: Respondents who explain at least the four major tools of CLTSH (transient walk, feces mapping, feces calculation, feces mobility chart and glass of water exercise).

## 3. Result

### 3.1. Socio-demographic characteristics of the study population

The interview sample comprised of 420 respondents, achieving the response rate of 99.5%. The age of the respondents ranged from 18 to 61 years, with the mean age of 37.25 (±7.7 SD) years (Table 1).

**Table 1:** Socio-demographic characteristics of population under study, 2014.

Variables (n = 420)		Frequency	Percent
**Age (year)**	Less than 30	71	16.9
	30–40	207	48.3
	Greater than 40	142	33.8
**Marital Status**	Single	38	9
	Married	341	81.2
	Divorced	24	5.7
	Widowed	15	3.6
	Separated	2	0.5
**Family Size**	Less than or equal to 4	232	55.2
	Greater than 4	188	44.8
**Monthly Income**	Less than 700	104	24.8
	700–1500	160	38.1
	1500–2000	89	21.2
	Greater than 2000	67	16
**Educational Level**	Illiterate	141	33.6
	Elementary (1–4)	123	29.3
	Junior (5–8)	77	18.3
	Secondary (9–10)	42	10
	Preparatory (11–12)	8	1.9
	Tertiary (12 +)	29	6.9
**Occupational Status**	Farmer	257	61.2
	Housewife	40	9.5
	Merchant	55	13.1
	Govt Employee	37	8.8
	Daily Labor	31	7.4
**Religion**	Orthodox	15	3.6
	Muslim	401	95.5
	Catholic	4	1

### 3.2. Factors associated with the CLTSH implementation

The findings revealed that 66% (279) of the respondents have knowledge of the CLTSH, of which 88.3% (246) obtained their knowledge from health extension workers. However, 14% (58) of the interviewees were not convinced that CLTSH is an important tool for eradicating open defecation, and a further 52% (218) were not aware of the ignition participatory rural appraisal tools (transient walk, faces mapping, faces calculation and faces mobility chart). As revealed in this study, only 22% of the respondents had a latrine prior to the introduction of CLTSH, and the remaining 78% constructed latrines after being triggered by CLTSH approach.

In addition, 68% (284) of the respondents had the view that the social mobilization techniques currently conducted in the study area have brought a change in the attitudes toward the practice of open defecation among the study population. Yet, 51% of the respondents perceived that the formed committees were not effectively working to achieve open defecation-free communities, even though 81% (339) of the respondents have been trained on the CLTSH.

Based on the current study findings, 86% (359) of the poor families (and 85% (357) of women) play a proportionate and active role in the implementation of CLTSH activities. Nearly 73% (305) of the respondents felt that all communities had been benefited equally from the CLTSH intervention. The support for poor families in terms of funding, skills, and materials required to construct a latrine is minimal. Moreover, 84% (353) of the respondents believed that CLTSH could also create social harmony. Regarding the continuous assessment of CLTSH, 63% (264) of the respondents concurred that there was a system of assessment by the community in regard to achieving the goals of CLTSH.

When asked if they owned a latrine, 89% (375) of the study participants responded affirmatively, and 79.5% (298) of this group indicated that their latrines were cleanly maintained. Yet, during the survey, 15% (62) of the respondents reported diarrheal cases among family members. The majority of the respondents decided to construct latrine after the introduction of the CLTSH approach in the study area. Therefore, it can be posited that this approach has enhanced latrine ownership in the study area, and this consequently reduces the prevalence of diarrheal cases.

The majority of the respondents stated that they used to defecate in their home surroundings (62%) or in the open field (31%) before the implementation of CLTSH. Because of the open defecation, 92% of the people in the study area lacked freedom and had been vulnerable to different fecal-oral diseases. According to the current findings, inconsistent use of latrine by all family members was observed, and only 47% (176) of the interviewees reported regular use of latrine by their entire family. But only 26.4% (99) of the respondents reported proper hand washing with soap and water after latrine use. Eighty-eight percent of the respondents reported lack of sanitary products such as cement slabs for the construction and maintenance of latrines. Conversely, 84% (353) of the study sample reported that there is a regular mobilization among the community to build a latrine, promoting consistent use and good hygiene practices (Tables 2–4).

**Table 2:** Selected variables measured by knowledge of CLTSH among study participants in Diretiyara district, Ethiopia, 2014.

Variable (n = 420)		Knowledgeable			Odds Ratio (95% CI)	
		Total	Yes	No	Crude	Adjusted
**Age in year**	< 30	71	55	16	0.50(0.26, 0.97)*	0.45(0.21,2.00)
	30–40	207	134	73	0.94(0.60, 1.47)	1.23(0.71, 2.13)
	≥ 40	142	90	52	1	1
**Educational status**	Illiterate	141	115	26	1.96(0.55, 6.97)	3.61(0.84,15.57)
	Elementary(1–4)	123	62	61	8.53(2.45, 29.65)*	18.99(4.48,80.54)*
	Junior (5–8)	77	56	21	3.25(0.89, 11.88)	6.46(1.49,28.08)*
	Secondary (9–10)	42	16	26	14.08(3.66,54.20)*	45.29(9.27,221.29)*
	Preparatory (11–12)	8	4	4	8.67(1.39, 54.03)*	25.03(2.74,228.68)*
	Above 12	29	26	3	1	1
**Occupational status**	Housewife	48	33	15	1.34(0.54, 3.25)	1.44(0.47,4.41)
	Merchant	42	30	12	1.17(0.46, 2.98)	1.23(0.38,3.95)
	Gov't worker	37	16	21	3.83(1.52, 9.64)*	4.14(1.28,13.40)*
	Farmer	246	165	81	1.43(0.71, 2.91)	1.81(0.75,4.36)
	Daily Labor	47	35	12	1	1
**Family size**	≤ 4	232	170	62	1	1
	>4	188	109	79	1.99(1.32,2.99)*	1.76(1.05,2.95)*
**Monthly income**	≤ 700	104	67	37	0.54(0.29, 1.00)	0.55(0.24,1.27)
	700–1500	160	111	49	0.43(0.24, 0.77)	0.43(0.20,0.90)
	1500–2000	89	68	21	0.30(0.15, 0.59)	0.27(0.12,0.60)
	>2000	67	33	34	1	1
**Accept CLTS**	Yes	362	256	106	1	1
	No	58	23	35	3.68(2.07, 6.52)*	8.28(4.05,16.94)*

Note

* = statistically significantly associated (the P value is less than 0.05).

**Table 3:** Selected variables as measured by latrine ownership among study participants in Diretiyara district, Ethiopia, 2014.

Statement (n = 420)		Latrine ownership		Odds Ratio (95% CI)	
		Yes	No	Crude	Adjusted
Accept the need of CLTSH program	Yes	336	26	1	1
	No	39	19	6.30 (3.20, 12.40)*	3.23 (1.20, 8.67)*
Know the steps of CLTSH	Yes	197	5	1	1
	No	178	40	8.85(3.42, 22.93)*	3.15(1.08,9.24)*
Practice of social mobilization	Yes	273	14	1	1
	No	102	31	5.93 (3.03, 11.59)*	2.39(1.04,5.49)*
Training of CLTSH program	Yes	323	16	1	1
	No	52	29	11.26 (5.72,22.16)*	4.20(1.81,9.78)*
Two-week diarrheal prevalence among family members	Yes	46	16	3.95 (1.99, 7.82)*	2.48(1.00,6.12)*
	No	329	29	1	1
System of reward and punishment to maintain the ODF status	Yes	135	02	1	1
	No	240	43	12.09 (2.88, 50.71)*	9.96(2.08,47.58)*
Promotion of latrine uses and good hygiene practice	Yes	325	28	1	1
	No	50	17	3.95(2.02,7.72)*	2.27(0.93,5.55)

Note

* = statistically significantly associated (the P value is less than 0.05).

**Table 4:** Attitude and perception towards CLTSH as measured by consistent latrine use among study participants in Diretiyara district, Ethiopia, 2014.

Statement (n = 375)		Consistent latrine use		Odds Ratio (95% CI)	
		Yes	No	Crude	Adjusted
Preferred open defecation due to unpleasant smell and heat from the latrine	Agree	151	155	0.58(0.34, 0.99)*	0.66(0.35,1.24)
	Disagree	25	44	1	1
Latrines are only intended for rich people	Agree	97	84	0.60 (0.40, 0.90)*	0.61(0.36, 1.03)
	Disagree	79	115	1	1
Latrines require periodic maintenance	Agree	160	193	1	1
	Disagree	16	6	0.31 (0.12,.81)*	0.29(0.10,0.87)*
Latrines built using local materials are affordable for low- income people	Agree	106	79	1	1
	Disagree	70	120	2.30 (1.52, 3.48)*	2.15 (1.25, 3.70)*
Open defecation is an ancestral practice passed down through generations	Agree	154	156	0.52 (0.30, 0.91)*	0.68 (0.35,1.32)
	Disagree	22	43	1	1
Cleanliness of the latrine	Yes	167	131	1	1
	No	9	68	9.63(4.63,20.03)*	10.24(4.69, 22.39)*
Knowledge of CLTSH	Yes	139	117	1	1
	No	37	82	2.63(1.66,4.17)*	3.15(1.81, 5.49)*
Acceptance of CLTSH	Yes	160	162	1	1
	No	16	37	2.28(1.22,4.27)*	2.45(1.14,5.27)*
Time latrine was constructed	Before CLTSH	27	54	1	1
	After CLTSH	149	145	0.49 (0.29, 0.82)*	0.53 (0.28,1.02)

Note

* = statistically significantly associated (the P value is less than 0.05).

### 3.3. Qualitative study associated with the implementation of CLTSH

Proper households’ latrine use yields health benefits and reduces the incidence of diarrheal disease. A sense of shame or disgust at the thought of ingesting excreta or being observed during defecating and, conversely, a sense of pride due to being able to use a latrine have emerged as the main motivating factors. Satisfaction with using latrine stems from privacy (e.g., during menstruation) and security against threats that arise from defecating in the open field (for example, snake and insect bites), as well as comfort (particularly at night and/or during rainy season).

During the FGD, a 45 years old community leader, a father of five children, respondent explained the situation as follows: “I realized that, my children *unknowingly consume their own and/or someone else’s feces through contaminated water*, *food and hand*. *They experienced diarrhea frequently; I was obliged to visit clinics 3–4 times a month*. *We also suffered from bad smell of faces in our surrounding*. *After CLTSH has been implemented in our village we got relief and librated from such burden*”

During observation, Unavailability of land, Local soil conditions (collapsing or requirement of lining), unavailability of water for latrine cleaning and hand washing in proximity to latrines were detected as shortcoming. Financial constraints, lack of materials and skills in the family were also raised as demotivating factors. Conversely, availability of technical advice and follow-up visits related to all aspects of latrine use and maintenance are considered as the key enabling factors. Perceptions of inconvenience, discomfort, moral issues and peer pressure associated with poor quality latrines were the causes many respondents cited for abandoning latrine use. During the FGD, a 37 years old woman, a mother of three children, respondent explained the condition: *“I have no doubt about the importance of CLTSH; I saw its benefit on my children’s health*. *Due to loose soil condition*, *I have planned to upgrade my latrine by using modern sanitation technology but I couldn’t obtain in the local market and the ones available are very expensive”*

## 4. Discussion

Diarrheal diseases remain the major cause of mortality and morbidity in low-income countries like Ethiopia [20]. Findings in the extant literature on CLTSH in Ethiopia indicated that a significant reduction in diarrheal incidences and nearly no acute watery diarrhea (AWD) incidence were achieved in the kebeles where CLTSH was implemented [15]. Thus, exploring the factors that influence the implementation of CLTSH to achieve open defecation-free (ODF) status is crucial.

The current study showed that 11% of the respondents practiced open defecation. This finding is promising compared to what the results of a study, conducted by Plan International Ethiopia in collaboration with the Institute of Water at the University of North Carolina, showed. As indicated in that study, 37% of the respondents practiced open defecation [21]. This result suggests that CLTSH implementation is effective and should be scaled up to other districts in the region. The implementation of CLTSH in the study area not only boosted latrine ownership and sanitation access, but also increased latrine cleanliness. Indeed, this was encouraging outcome of the approach in the reduction of the fecal-oral disease. This finding is supported by the results yielded by a study conducted by Alzua et al. in Mali [22].

In this study, 15% of the respondents reported to have experienced diarrheal cases among family members in the last two weeks during the study period. This is lower than what was shown by the findings of a study conducted in Jimma zone Kersa district- where the overall two weeks period prevalence of diarrhea in the area where CLTSH was implemented was 19% [23]. This indicates that there is a better implementation of CLTSH among the current study population. In the current study, the participants’ knowledge of CLTSH was significantly associated with age (being less than 30), educational status, being a government worker, family size and acceptance of the importance of CLTSH, as was revealed by the bivariate and multivariate analysis. Respondents who did not accept the CLTSH approach [AOR = 8.28; 95% CI 4.05, 16.94] were eight times more likely not to have knowledge about CLTSH compared to their counterparts.

In the current study, weak follow-up system to aggregate the data, track progress and assist people to move up the sanitation ladder (unimproved—basic—improved sanitation), poor commitment of health extension workers and facilitators, triggering of CLTSH without adequate preparation, lack of effective social mobilization techniques, inadequate participation of women and support of poor families, unavailability of sanitation technology to access a more advanced and safer form of sanitation, and lack of periodic training for in-depth understanding of the ignition participatory rural appraisal tools, might be impeding factors against the effective implementation of CLTSH and sustainability in the study area. This finding is supported by studies conducted by Mengistu [11], Kappauf [24] and Bongartz et al [25]; and these factors were also validated by discussant during focus group discussion.

Using both bivariate and multivariate analysis latrine ownership was found to be significantly associated with acceptance of CLTSH, knowledge of the steps of CLTSH, the practice of social mobilization, training about CLTSH, two-week diarrhea prevalence, reward and punishment system, and promotion of latrine use and good hygiene practice. Inadequate promotion of latrine use and lack of good hygiene practice [COR = 3.95; 95% CI 2.02, 7.72] were three times more likely to have been causes for not to have latrine compared with their counterparts. Moreover, the occurrence of diarrhea was also found to be significantly associated with the extent of latrine ownership in bivariate [COR = 3.95; 95% CI 1.99, 7.82] and multivariate [AOR = 2.48; 95% CI 1.00, 6.12] analysis. This might indicate that not only is the availability of the latrine important but its consistent and proper use is also vital. Hence, further effort is needed to bring about behavioral change for the sustainable and firm implementation of CLTSH in the study area.

Attitude and perception parameters are significantly associated with consistent latrine utilization. Respondents who believed that latrine is intended only for rich people [COR = 0.60; 95% CI 0.40, 0.90] were 60% less likely to use the latrine consistently. Respondents who have unclean latrine [AOR = 10.24; 95% CI 4.69, 22.39] were 10 times less likely to use the latrine consistently, and eighty-two percent of the respondents believed that, "Open defecation is preferred due to the unpleasant smell and heat from the latrine". This indicates that the cleanliness of the latrine should be maintained and effective follow up and boosting of the system by the community to the upper sanitation ladder is crucial. Furthermore, the respondents who agreed on the idea that "Open defecation is an ancestral practice passed down to generations" are 52% less likely to use the latrine consistently. This indicates that to end open defecation, more is needed to be done on the behavioral change of the respondents towards sanitation and hygiene.

## 5. Conclusion

In this study, 34% of the respondents were found to have inadequate knowledge about CLTSH and 11% of them reported to have practiced open defecation. Poor follow up, lack of effective social mobilization and full participation of a significant portion of the society such as women, poor families and children were observed to be significant causes for open defecation.

This study also showed that CLTSH has increased the extent of latrine ownership, cleanliness, and its utilization. On the other hand, lack of in-depth periodic training, promotion of latrine use and hygienic practice were reported. This study indicated that 14% of the respondents did not still accept this approach as a means to end open defecation in their village. This could act as a bottleneck for the community to scale up sanitation ladder. Moreover, this study shows that there is a fundamental misconception about CLTSH among a study population, which is a crucial factor for sustainable behavioral change towards their sanitation and hygiene practice.

## Supporting information

S1 Table Original data of statistical analysis performed for Tables 1–3. (SAV)

S2 Table Original data of statistical analysis performed for Table 4. (SAV)

S3 Table Questionnaire:-Original Language Version. (DOCX)

S4 Table Questionnaire:-English Language Translated Version. (DOCX)

## Abbreviations

CLTSH: Community-Led Total Sanitation and Hygiene
FGD: Focus Group Discussion
SDG: Sustainable Development Goal
UN: United Nation
ODF: Open Defecation Free
WASH: Water, Sanitation, and Hygiene
