# Supplementary material for: Assessment of the implementation of community-led total sanitation, hygiene, and associated factors in Diretiyara district, Eastern Ethiopia
Source: PLoS One. 2017 Apr 13;12(4):e0175233. doi: 10.1371/journal.pone.0175233 (PMC5390995; doi:10.1371/journal.pone.0175233)
Supplement: S3 Table — (DOCX) [file pone.0175233.s003.docx]

**Local Language Version: Oromifaa Version of Questionnaire**

**Univarsitii Haamaayaa tti koleggii saayinsii fayyaa**

**Faaydaan qooranichaa:** Faayidaan qoranichaas dhukkuba qulquliina naanno dhaaburra Kankan eettissuf bu,aa gudaa qabaa.kanaarrati hundaye karroora baasuf nifaayyada.

1. Maqaa Aanaa………………………….. Maqaa Ganda:……………………
2. Maqaa abba raga funaanu ……lakkofsa hojatu HEWs ganda kana kessa --------

**Kutaa 1: Gaafilee Dhimma Jireenyaa Fi Hawaasummaa abbaa Raga Keenu**

| **lakko** | **Gaafilee** | **Deebi gaafilee** | **daarbi** | **Code** |
| --- | --- | --- | --- | --- |
| 101 | Urmiin kee waggaa guutuudhaan meeqa? | -------/wagga |  | AI101 |
| 102 | Sadarkaan barumsa kee hangami? | 1. Barumsa hinqabu  2. Sadarkaan 1ffa (1-4)  3. Sadarkaan Juniori (5-8)  4. Sadarkaan 2ffa (9-10)  5. Sadarka preparatory (11-12)  6. Sadarkaan oolaanna( 12 +) |  | ES102 |
| 103 | Hojiin maali? | 1. Haadha manaa ti  2.Daaldaaltuu  3. Hojii mootummaa ti  4.Hojjata guuyaa  5.Qootte bulla  6.Kan biraa (ibsaa) |  | OC103 |
| 104 | Saalli daa’imicha gaafiin kun guutamuufi haa ibsamu | 1.Dhiira  2.Dhalaa |  | SX104 |
| 105 | Amanntiin kee kami? | 1. Ortoodoxii  2. Musliima  3. Caatooliki  4. Prootestaantii  5. Kan Biraa(Ibsaa) ----------- |  | RE105 |
| 106 | Dhima heerumaa | 1. Hinfuune.  2. Fudhera  3. Addan banerra  4. Abban mana dueera  5. Qoba qoba jirana |  | MS 106 |
| 107 | Baayyinni maattii warra kanaa waligaalti meeqa? | 1.Tokko  2.Lamma  3.Saadii  4.Afuur  5. Shani fi Shani Ole |  | RS107 |
| 108 | Galiin warra kanaa hangami tti shalagama? | 1. Less than 700  2.700 - 1500  3.1500 - 2000  4.Greater than 2000 |  | MI 108 |

**Kutaa II: Gaafiilee sadarkaa mana irati dhimma CLTSH illalatu**

| **lak** | **Gaafilee** | | **Deebi gaafilee** | **daarbi** | Code |
| --- | --- | --- | --- | --- | --- |
| 109 | Dhimma CLTSH oddefaano/bekumsa qabdaa? | | (a)eyyee  (b)hinqabu |  | ES109 |
| 110 | lakko109,Yoo eyyee jette, oddefaano egnura argate? | | (i)qondaaltota fayara  (ii) leenjii edile ra  (iii)tojatota NGO ganda ra  (v)hoggentota siyaasaa  (vi)kan bira ibsi:…………… |  | AW110 |
| 111 | CLTSH berbachisaadh jete niyaada? | | (a) eyyee [ ]  (b) lakki [ ] |  | FN111 |
| 112 | lakko111, yoo lakki jette, | | maalif? ebsi |  | WE112 |
| 113 | Waaye CLTSH kana, egnut gandaa kanat fide? | | (a) hoggantota eggumsa fayaa  (b) hoggantota NGO  (c) hawwasuma kesa namootuma tokko tokko  (d) kan bira ibsi |  | DC113 |
| 114 | steepi CLTSH maalmaal akka tahan ni beytaa? | | (a) eyyee [ ]  (b) lakki [ ] |  | ST 114 |
| 115 | lakko114,Yoo eyyee tahhe, hujin armaan gaaddi nihojetaamani? | | (a)Pre- triggering  (i) eyyee (ii) lakki  (b)Triggering  (i) eyyee (ii) lakki  (c )Post-triggering  (i) eyyee (ii) lakki  (d)Scaling up & going beyond CLTSH  (i) eyyee (ii) lakki |  | TA115 |
| 116 | Straatejonii lakkofssa 115  irraat caqasaaman naannno  kessanitii nihojjaatamu? | | (a) eyyee [ ]  (b) lakki [ ] |  | PR116 |
| 117 | Yoo gaafi lakkofsa 116 eyyee yootah, hojjin kun nihojaatamu? | | (a) ganda kessa sosochou  (a) eyyee [ ]  (b) lakki [ ]  (b) iddo udaani maapi godhu  (a) eyyee [ ]  (b) lakki [ ]  (c) udaan kalkuulati godhu  (a) eyyee [ ]  (b) lakki [ ]  (d) udaan hala itibitinahu  (a) eyyee [ ]  (b) lakki [ ] |  | AT117 |
| 118 | Yoo gaafi lakkofsa 117 lakki yootah, haali maal fakkaata? | | (i)akka manaati………………………..  (ii) akka dhufati………………………  (iii)akka nnaannoti:……………………… |  | SC118 |
| 119 | Qulqulina naanno eguf  soshaal mobilayzation nijiira? | | (a) eyyee [ ]  (b) lakki [ ] |  | SM119 |
| 120 | Yoo gaafi lakkofsa 119 eyyee yootah, techniques maalfa akathaan ibsi: | | (i)…………………………………………….  (ii)…………………………………………….  (iii)……………………………………………. |  | DT120 |
| 121 | Qulqulina naanno egurati  soshaal mobilayzation bua  gaari nifida jette yaadu ? | | (a) eyyee [ ]  (b) lakki [ ] |  | ST121 |
| 122 | Yoo gaafi lakkofsa 121eyyee yootah, qabatmaan jijjirafiidu ibsii | | ibsi |  | WC122 |
| 123 | Qulqulina manaafi naanno  egufi manafinchani ijjaruf  kakaswa ummeta nijira? | | (a) eyyee [ ]  (b) lakki [ ] |  | CA123 |
| 124 | Yoo gaafi lakkofsa 123 eyyee  yootah, techniques maal  fayadamtu ummeta kakasuf: | | (i)…………………………………………  (ii)…………………………………………(iii)……………………………………… |  | TM124 |
| 125 | Committeen mataduredhan  hujikan addemsu  gurmaayeraa? | | (a) eyyee [ ]  (b) lakki [ ] |  | UC125 |
| 126 | Yoo gaafi lakkofsa 125 eyyee  yootah, korren kun maalmalfa hojatu?: | | (i)………………………………………  (ii)………………………………………  (iii)…………………………………………  (iv)……………………………………… |  | ZA126 |
| 127 | Kannadura waaye CLTSH lenjii argate bektu? | | (a) eyyee [ ]  (b) lakki [ ] |  | AT127 |
| 128 | Yoo gaafi lakkofsa 127 eyyee  yootah, lenjiinkun maalmalfarati xiyyefata? | | 1. Qulqulina mana finchanirati   [ ]  (ii) mnajimanti dhima CLTSH [ ]  (iii) CLTSH sala hunduma hirmesurati [ ]  (iv) qulqulina mana gnata irati  [ ]  (v) dhima hala stove foyesurati  [ ]  (vi) kanbira ebsa  [ ] |  | TP128 |
| 129 | Namoni kabegna hinqabne  hojirati walqita hirmatu? | | (a) eyyee [ ]  (b) lakki [ ] |  | PF129 |
| 130 | Yoo gaafi lakkofsa 129 lakki  yootah | | Maalif,yaebsaamu: |  | EP130 |
| 131 | dubaartooni hojirati walqita  hirmatu? | | (a) eyyee [ ]  (b) lakki [ ] |  | WP131 |
| 132 | Yoo gaafi lakkofsa 131 eyyee  Yootah,issan kun garre kaamirati? | | (i) maati dureesara [ ]  (ii) maatihiyesaara [ ]  (iii) maati barateera [ ]  (iv) hundumtu walqita hirmaatu[ ] |  | TF132 |
| 133 | Umaani kun CLTSH ra walqita fayadema jira jetee yaada? | | (a) eyyee [ ]  (b) lakki [ ] |  | BE133 |
| 134 | Yoo gaafi lakkofsa 133 lakki  yootah | | Ibsu dandea? ibsi:  ……………………………………… |  | EB134 |
| 135 | Teknoologin gahumsaan argatama umaata haarkaqaaleyi fi laafa hinqabnef tajaajilu mana finchani jaruf ni jiraa? | | (a) eyyee [ ]  (b) lakki [ ] |  | AT135 |
| 136 | Yoo gaafi lakkofsa 135 eyyee  Yootah? Maalisaan ibsi? | | ibsi.…………………………… |  | WO136 |
| 137 | Namooti harkaqaleyii hali iti qarqaraman jira? | | (a) eyyee [ ]  (b) lakki [ ] |  | SP137 |
| 138 | Yoo gaafi lakkofsa 137 eyyee  Yootah? Halakamin qarqareman: | | (i) gargarsa mesha [ ]  (ii) gargarsa qarshi [ ]  (iii) gargarsa ogeesa [ ]  (iv) hundumanu [ ] |  | SP138 |
| 139 | | Hirmanan CLTSH kun maal ummu dendaya? | (a)waligaltee hawasa [ ]  (b)waldhaabu hawaasa [ ]  (c )huma hinumu [ ] |  | CP139 |
| 140 | | Haal qormaata iti gegesitu fi  proograamich haalagaridhan  adeemsa akka jiru it hoataan  jirat? | (a) eyyee [ ]  (b) lakki [ ]  (c) hinbeku[ ] |  | SA140 |
| 141 | | Yoo gaafi lakkofsa 140 eyyee  Yootah? Haala kamin? | ibsii……………………………………………………………………..………………… |  | HE141 |
| 142 | | Mana finchaani qabdaa? | (a) eyyee [ ]  (b) lakki [ ] |  | HL142 |
| 143 | | Yoo gaafi lakkofsa 142 eyyee  Yootah? Toorbaan darbe leman kessa namni garakaasadhan dhukubame jira? | (a) eyyee [ ]  (b) lakki [ ] |  | YD143 |
| 144 | | Yoo gaafi lakkofsa 142 lakki  Yootah? Toorbaan darbe leman kessa namni garakaasadhan dhukubame jira? | (a) eyyee [ ]  (b) lakki [ ] |  | ND144 |

| 145 | | Yoo gaafi lakkofsa 142 lakki  Yootah? Sababni isa maali? | (a) lafaa hinqabu  (b) qotuf laafni dhaga/cimaadha  (c) lafni chirech  (d) gaatin isaa cimmaadha  (d) haala itiqotaan hinbekuu | |  | WR145 |
| --- | --- | --- | --- | --- | --- | --- |
| 146 | | Yoo gaafi lakkofsa 142 eyyee  Yootah? Akkaakun isaa kami? | (a) Pit laatriine  (b) mana finchani kan qilesaa basaa qabu  (c) mana finchani kan bishan qabu | |  | TL146 |
| 147 | | Yoo gaafi lakkofsa 142 eyyee  Yootah? Yoomira jelqqabde fayyadamu egaltee? | (i) CLTSH osso dhufin duraa [ ]  (ii) CLTSH egga dhufe boda [ ] | |  | HL147 |
| 148 | | Yoo gaafi lakkofsa 142 eyyee  Yootah? Egga hojete boda suphana gotefi nibekta? | (a) eyyee [ ]  (b) lakki [ ] | |  | UG148 |
| 149 | | Yoo gaafi lakkofsa 148 lakki  Yootah? maalif? | (i) qarsh dhabudhan [ ]  (ii) lafaa dhabudhan [ ]  (iii) addara kan kaen [ ]  (iv) bekumsa dhabura kan kae[ ]  (v) kanbira ibsi… | |  | WP149 |
| 150 | | Mana finchani jaarudhaf rakko maalfa siqunaame? | (a)…………………………………………  (b)…………………………………………  (c ……………………………………….. | |  | FC150 |
| 151 | | Yoo gaafi lakkofsa 142 lakki  Yootah? Esaati fayyadamtuu? | (a) kararati [ ]  (b) naanno manaa[ ]  (c ) dagalaa kessa [ ]  (d) hunduma [ ] | |  | DB151 |
| 152 | | Yerro manaa finchaani hinqabne saan raako malfa siquname? | (a)……………………………………………………………………………………  (b)………………………………………………………………………………………  (c )……………………………………….. | |  | FL152 |
| 153 | | Maatin maanakesaan hunduntu maana finchani nifayaadamu? | (a) eyyee [ ]  (b) lakki [ ] | |  | RU153 |
| 154 | | Yoo gaafi lakkofsa 153 lakki  Yootah? Maana kesaa egnu kanhinfayyadamne? | (a) abba maanar [ ]  (b) hadha maana [ ]  (c ) jolle dubartoota[ ]  (d) jolle dhira [ ]  (d) maalif hinfayyadamne?  ………………………………………… | |  | UL154 |
| 155 | | Qulqulina maana finchani niegdu? (ijjaan laali) | (a) eyyee [ ]  (b) lakki [ ] | |  | MC155 |
| 156 | | Yoo gaafi lakkofsa 155 eyyee  Yootah? Maalin egdu? | ibsi…………………………….  …………………………………… | |  | PE156 |
| 157 | | Maana finchani boda harka ni dhiqata? | (a) eyyee [ ]  (b) lakki [ ] | |  | HW157 |
| 158 | | Yoo gaafi lakkofsa 157 eyyee  Yootah? Maalin dhiqata? | (i) bishan qofaan [ ]  (ii) daara fi bishanin[ ]  (iii) saabunafi bishanin[ ]  (iv) hunduma naafayyadame[ ] | |  | HW158 |
| 159 | | Maana finchani jaarachuf maal fayyadamta? | | (a) boolof:………………………  (b) maana boologubaf……… |  | YU159 |
| 160 | | Mesha maanafinchani itijaraten  nannokessanit niargamaa ? | | (a) eyyee [ ]  (b) lakki [ ] |  | SP160 |
| 161 | | Yoo gaafi lakkofsa 160 eyyee  Yootah? | | (i) egga CLTSH dhufe duraa [ ]  (ii) egga CLTSH dhufe booda [ ] |  | BI161 |
| 162 | | Namni tokko yoo naano balessa haali iti adaabamu ykn bedhafamu jira? | | (a) eyyee [ ]  (b) lakki [ ] |  | SR162 |
| 163 | | Yoo gaafi lakkofsa 162 eyyee  Yootah? Maal isaan ibsi? | | (a)…………………………………………  (b)…………………………………………  (c )…………………………………….. |  | WT163 |
| 164 | | Dhima kanaraat mariachuf waalghi niqabdu jirrata ganda wajine? | | (a) eyyee [ ]  (b) lakki [ ] |  | SO164 |
| 165 | | Yoo gaafi lakkofsa 164 eyyee  Yootah? Akkamit ilaalta? | | (i) baaye effective dha [ ]  (ii) Effectivedha  (iii) effective mitte [ ] |  | ET165 |
| 167 | Dhima kannarat yaada mal qabda? | | Iftominan ibsi |  | IS167 |  |

**Kuta III: Gaafille ammanti ummetarati qiyyeefate gafataamun waayye qulqulinaa naanorati qiyefate.**

Gaafille ammanti ummetarati qiyyeefate gafataamuu,waae qulqulinaa naano siiridhan gegesuu ykn gegesu dhabu iraata kaan qiyyefate.

| **Lk** | **Gaafille ammanti ummetarati qiyyeefate** | **Itiin amaana** | **Itin**  **amaanuu** |
| --- | --- | --- | --- |
|  | Dagala kesa udaanuun bayaa namaate toolaa, baayye seeftis niqaba,fayyadhaf gaaridh. |  |  |
|  | Dagala keesaa udaanu nanfilaadha maalifi foolefi owena xajaya manaa finchani kessa bahu saaniraa bilisaa taha |  |  |
|  | Maanifinchaani dureesa qofaf barbachisa nuti isaa qotudhan duresaa ufgoodhu barbaachisa miti. Wan itqonu qarshi qabnuule hiyyesaaf barbachisa miti. |  |  |
|  | Maanifinchaani tooko jaarmnaan yeroo yeroo suphaana nibarbaadae |  |  |
|  | Maanifinchal meshaa naanokesaat argamura jaraame harkaaqaleyif baaye gaaridh |  |  |
|  | Naano manaa finchani want akka jinni niargamuu knaafu maani finchan barbaachisa miti. |  |  |
|  | Maana finchani kessat udaanun umrii namaa nigababsaa |  |  |
|  | Baalenaarati udaanun wantaa abooti keegnera nudhufe knaafu itifufu qabnaa |  |  |
|  | Baalenaarati udaanun baaye namaati toola |  |  |

**Kuta IV: Gaafille Matadureef Tahan Mari Abbooti Bulchinsa Wajin Godhamuf**

1. Waaye CLTSH kun kosteffective dha haala biro wajin medale hogalalu?
2. Dhimi kun hala akamin akstanshini fayyedhan kenama jira?
3. Waaye CLTSH kaan ool gudisuuf barbachisadha ykn raakon isaa wajin jiru maal maalfadha?
4. Issa kan gudisuf gadifagegnan karoora niqabdul?
5. Ummani hundumtu sirridha nihirmaatu?
6. Dhima kanaarat mootuman siridhan hirmaana niqaba?
7. Qulqulina naanno egsisuf Qooni duubartoota fi joolle mal tauu dandaa?
8. Qulqulina naanno egsisuf raakkooni jiraan maalmaal taauu dandauu?
9. Uumani kun Waayee CLTSH qabaatama dhan hojjete itti fufu nidanda jete yada?
10. Yaadaa keetit naamota hiyeeyyi fi mana finchani qotuf laafa hinqabne maalhaagodhamu jete yaadaa?
