# Supplementary material for: Assessment of the implementation of community-led total sanitation, hygiene, and associated factors in Diretiyara district, Eastern Ethiopia
Source: PLoS One. 2017 Apr 13;12(4):e0175233. doi: 10.1371/journal.pone.0175233 (PMC5390995; doi:10.1371/journal.pone.0175233)
Supplement: S4 Table — (DOCX) [file pone.0175233.s004.docx]

**Appendix 1: Questionnaire: English Language Translated Version**

Haramaya University College of Health and Medical Science Young Faculty Research Grant on: Assessment of the Implementation of Community-Led Total Sanitation, Hygiene and associated factors in Diretiyara district, Eastern Ethiopia.

1. Name of sub-kabele…………………………..Name of Ganda:……………………
2. Name of interviewer:………………………No of Health extension worker per kabale -----

**PART I: Socio-Demographic Characteristic**

| **S/No** | **Questions** | **Alternatives** | **Skip** | **Code** |
| --- | --- | --- | --- | --- |
| 101 | Age of interviewee | -------/in Year/ |  | AI101 |
| 102 | Educational status | 1.Illitrate  2. Elementary (1-4)  3. Junior (5-8)  4. Secondary (9-10)  5. Preparatory (11-12)  6. 12 + |  | ES102 |
| 103 | Occupation | 1. House wife  2.Merchant  3.Government employee  4.Daily labor  5.Farmer  6.Other |  | OC103 |
| 104 | Ethnicity of the interviewee | 1. Oromo 2. Amhara 3. Gurage 4. Harari 5. Other (specify) |  | RS104 |
| 105 | Religion | 1. Orthodox  2. Muslim  3. Catholic  4. Protestant  5. Other specify----------- |  | RE105 |
| 106 | Marital status | 1. Single…………………...  2. Married  3. Divorced  4. Widow  5. Separated |  | MS 106 |
| 107 | Family size | 1.One  2.Two  3.Three  4.Four  5. Five and above |  | RS107 |
| 108 | Monthly income level | 1. Less than 700 2. 700 - 1500 3. 1500 - 2000 4. Greater than 2000 |  | MI 108 |

**PART II: Household Questionnaire to Assess CLTSH**

| S/N | | Questions | Alternatives | | Skip | Code |
| --- | --- | --- | --- | --- | --- | --- |
| 109 | | Do you know about CLTSH program? | (a)Yes  (b) No | |  | ES109 |
| 110 | | If yes, for Q109 source of information about CLTSH program? | (i)Local health Staff  (ii)Award during formal training  (iii)Local NGO  (v)Political Leaders  (vi)If any other, please explain:…………… | |  | AW110 |
| 111 | | Do you accept need of CLTSH program? | (a) Yes [ ]  (b) No [ ] | |  | FN111 |
| 112 | | If no for Q111 | why? explain | |  | WE112 |
| 113 | | Who designed the CLTSH Program in your village? | (a) Local health office  (b) Local NGO  (c) Community Members  (d) other specify | |  | DC113 |
| 114 | | Do you know the steps of CLTSH? | (A)Yes  (B)No | |  | ST 114 |
| 115 | | If yes, for Q114 had the following phase been applied? | (a)Pre- triggering  (i) Yes (ii) No  (b)Triggering  (i) Yes (ii) No  (c )Post-triggering  (i) Yes (ii) No  (d)Scaling up and going beyond CLTS  (i) Yes (ii) No | |  | TA115 |
| 116 | | Have the Ignition Participatory  Rural Appraisal (PRA) tools been used as intervention strategy in your community? | (A) Yes [ ]  (B) No [ ] | |  | PR116 |
| 117 | | If yes, for Q 116 had the following tools been applied? | (a) Transient walk  (i) Yes [ ]  (ii) No [ ]  (b) Feces Mapping  (i) Yes [ ]  (ii) No [ ]  (c) Feces Calculation  (i) Yes [ ]  (ii) No [ ]  (d) Feces Mobility Chart/flow diagram  (i) Yes [ ]  (ii) No [ ]  (e) Glass of water exercise  (i) Yes [ ]  (ii) No [ ] | |  | AT117 |
| 118 | | If no, for Q117 what was the situation of your community before ignition PRA activities? | 1. At House Hold ……………………..   (ii) personal:………………………………  (iii) School ( If )………………………….  (iv) Environmental:………………………. | |  | SC118 |
| 119 | | Have the social mobilization  programs been launched  to achieve No Open defecation  Communities? | (a) Yes [ ]  (b) No [ ] | |  | SM119 |
| 120 | | If yes, for Q119 please explain  the different techniques: | (i)…………………………………………….  (ii)…………………………………………….  (iii)……………………………………………. | |  | DT120 |
| 121 | | Are the social mobilization techniques effective in bringing change in the attitude and practice of the people towards sanitation and hygiene | (a) Yes [ ]  (b) No [ ]  (c) Don't know [ ] | |  | ST121 |
| 122 | | If yes, for Q121 what are the changes?………. | explain | |  | WC122 |
| 123 | | Has the community appointed a health motivator to mobilize the community to maintain household and environmental sanitation and to construct latrine and make its proper use? | (a) Yes [ ]  (b) No [ ] | |  | CA123 |
| 124 | | If yes, for Q123 what  techniques they adopt to  mobilize the people: | (i)…………………………………………….  (ii)…………………………………………….(iii)……………………………………………. | |  | TM124 |
| 125 | | Have the users' committees  been formed in CLTSH  communities and whether  they have played an active  role in the project? | (a) Yes [ ]  (b) No [ ] | |  | UC125 |
| 126 | | If yes, for Q125 what are their activities: | (i)…………………………………………….  (ii)…………………………………………….  (iii)…………………………………………….  (iv)……………………………………… | |  | ZA126 |
| 127 | | Has there been any training in CLTSH program for the capacity building of the community? | (a) Yes [ ]  (b) No [ ] | |  | AT127 |
| 128 | | If yes, for Q127 are those training programs related to? | 1. Construction of hygienic latrine   (ii) management training related to CLTSH  (iii) Gender participation on CLTS  (iv) Kitchen gardening training  (v) Improved cooking stove use training  (vi) All of the above | |  | TP128 |
| 129 | | Do the poor families, have  proportionate and active role  in implementation of CLTSH activities? | (a) Yes [ ]  (b) No [ ] | |  | PF129 |
| 130 | | If no, for Q129 | Why, explain: | |  | EP130 |
| 131 | | Do the women have  proportionate and active role  in implementation of CLTSH activities? | (a) Yes [ ]  (b) No [ ] | |  | WP131 |
| 132 | | If yes, for Q131 are they from? | (i) Rich families [ ]  (ii) Poor families [ ]  (iii) Educated families [ ]  (iv) all are equally participate [ ] | |  | TF132 |
| 133 | | Do you feel that all community benefited equally from CLTSH interventions? | (a) Yes [ ]  (b) No [ ] | |  | BE133 |
| 134 | | If no, for Q133 | Please explain how they will be benefited:  ……………………………………… | |  | EB134 |
| 135 | | Are there adequate technological options for the construction of cheap latrine to suit the economic conditions of poor and landless households? | (a) Yes [ ]  (b) No [ ] |  | | AT135 |
| 136 | | If yes, for Q135 what are the options? | Please explain.…………………………… |  | | WO136 |
| 137 | | Was there any support for poor people? | (a) Yes [ ]  (b) No [ ] |  | | SP137 |
| 138 | | If yes, for Q137 was it in the form of: | (i) Material Support [ ]  (ii) Financial Support [ ]  (iii) Technical Support [ ]  (iv) All of the above [ ] |  | | SP138 |
| 139 | | Does the CLTSH program create? | (a) Social harmony [ ]  (b) Conflict [ ] |  | | CP139 |
| 140 | | Is there a system of  assessment by the community  in regard to achieving the  goals of CLTSH | (a) Yes [ ]  (b) No [ ] |  | | SA140 |
| 141 | | If yes, for Q140 how? | explain……………………………………………………………………. |  | | HE141 |
| 142 | | Do you possess latrine? | (A) Yes [ ]  (B) No [ ] |  | | HL142 |
| 143 | | If yes, for Q 142, does had diarrhea in the last 2 weeks among family members? | 1. Yes ------------------------[ ] 2. No --------------------------[ ] |  | | YD143 |
| 144 | | If No, for Q 142, does had diarrhea in the last 2 weeks among family members?? | 1. Yes -------------------------[ ] 2. No --------------------------[ ] |  | | ND144 |
| 145 | | If No, for Q 142 what was the reason? | (a) No land to construct it [ ]  (b) Ground is too hard/rocky [ ]  (c) Ground is too sand/unstable [ ]  (d) Cost of building is unaffordable [ ]  (d) No technical skill to build it [ ] |  | | WR145 |
| 146 | | If yes, for Q 142 what type of latrine you have? | (a) Pit latrine [ ]  (b) VIP [ ]  (c) Water flush latrine [ ]  (d) Other specifies [ ] |  | | TL146 |
| 147 | | If yes, for Q 142 when do construct the latrine? | (i) Before CLTS approach [ ]  (ii) After CLTS approach [ ] |  | | HL147 |
| 148 | | If yes, for Q142 have you upgraded the latrine since construction? | (a) Yes [ ]  (b) No [ ] |  | | UG148 |
| 149 | | If no, for Q148 what are the problems? | (i) Financial [ ]  (ii) Landlessness [ ]  (iii) Cultural [ ]  (iv) Unawareness [ ]  (v) If others, please explain:……………………………………………………………………………… |  | | WP149 |
| 150 | | What factors compelled you to construct latrine? | (a)……………………………………………  (b)………………………………………… |  | | FC150 |
| 151 | | Where did you defecate before CLTSH Program? | (a) Along road side [ ]  (b) Home surrounding [ ]  (c ) Open field [ ]  (d) All of the above [ ] |  | | DB151 |
| 152 | | What problems did you face when you had no latrine? | (a)…………………………………  (b)…………………………………………  (c )……………………………………….. |  | | FL152 |
| 153 | | Are all the family members regularly using latrine constructed in your house? | (a) Yes [ ]  (b) No [ ] |  | | RU153 |
| 154 | | If no, for Q153 who don't regularly use the latrine? | (a) father [ ]  (b) mother [ ]  (c ) girls [ ]  (d) boys [ ]  (d) What are the reasons no use?  …………………………………………… |  | | UL154 |
| 155 | | Have you maintained cleanliness of your latrine? (observe) | (a) Yes [ ]  (b) No [ ] |  | | MC155 |
| 156 | | If yes, for Q155 how? | Please explain…………………………….  ………………………………………………………………………… |  | | PE156 |
| 157 | | Do you wash your hands after defecation? | (a) Yes [ ]  (b) No [ ] |  | | HW157 |
| 158 | | If yes, for Q157 what detergent used? | (i) with Water only [ ]  (ii) Ash and water [ ]  (iii) Soap and water [ ]  (iv) All of the above [ ] |  | | HW158 |
| 159 | | What have you used in construction of your latrine? | (a) At Pit level:………………………  (b) Superstructure:………………… |  | | YU159 |
| 160 | | Are the Sanitary products used  for construction materials like  cement slab available locally  for the construction, operation  and maintenance of latrine ? | (a) Yes [ ]  (b) No [ ] |  | | SP160 |
| 161 | | If yes, for Q160 | (i) Before the Intervention [ ]  (ii) After the Intervention [ ] |  | | BI161 |
| 162 | | Is there any system of reward and punishment to maintain the open defecation free status? | (a) Yes [ ]  (b) No [ ] |  | | SR162 |
| 163 | | If yes, for Q 162 what are they? | (a)………………………………………  (b)………………………………………  (c )……………………………………… |  | | WT163 |
| 164 | | Do you have the system of organizing coordination meetings/mass gatherings, in your cluster/community to build latrines and promote good hygiene practices? | (a) Yes [ ]  (b) No [ ] |  | | SO164 |
| 165 | | If yes, for Q164 how effective are they? | (i) Most effective [ ]  (ii) Effective  (iii) Not effective [ ] |  | | ET165 |
| 167 | If you have any idea for the success of the program can you explain? | Please explain |  | | IS167 |  |

**Part III: Attitude and perception related factors associated with CLTSH**

Attitude and perception questions related to practice open defecation and their resistance to changing their open defecation practices

| **Sn** | **Attitude and perception related factors** | **Agree** | **Disagree** |
| --- | --- | --- | --- |
|  | Feel that defecating in the bush offers more dignity, privacy, safety, comfort and wellbeing |  |  |
|  | Preferred open defecation because they believed it prevented from unpleasant smell and ‘heat’ from the latrine |  |  |
|  | Latrines are only intended for rich people, and you should not compare yourself to them and build latrines, even if you can afford the cost. |  |  |
|  | Traditional latrines designs require periodic maintenance |  |  |
|  | Latrines built using local materials are more affordable for poor people |  |  |
|  | Toilets are surrounded by evil spirits and therefore should be avoided |  |  |
|  | Defecating in latrines shortens life span of people |  |  |
|  | Open defecation is seen as an ancestral practice passed down through generations |  |  |
|  | Comfortable with the practice of open defecation |  |  |

**Part IV: Guidelines for Focus Group Discussion (FGD) with** **community leader***,* **Health Extension Workers (HEW), Health Post Staff and Health Bureau staffs**

1. To what extent is the CLTSH approach been cost effective as compared to other approaches in terms of cost per beneficiary and funds spent on hardware and software?
2. What advantages acquired after the introduction of CLTSH in your village and of How far are CLTSH tools applied by HEWs been more effective than the traditional practices?
3. Is CLTSH an effective approach to take to scale and if so what are the barriers in achieving this?
4. Is there Detailed Action Plan with adequate provision of capacity building of the community to make the CLTSH successful?
5. Do every community members have commitment to declare ODF community?
6. Is there political commitment for the program from local government officials and other local leaders?
7. What has been the role of women and children in achieving ODF community?
8. What are the barriers in achieving ODF community?
9. Do you think your community to be capable enough to sustain CLTSH?
10. In your feeling what needs to be done for poor and landless people in your community?
